# Polycomb Group Genes *Psc* and *Su(z)2* Maintain Somatic Stem Cell Identity and Activity in *Drosophila*

**DOI:** 10.1371/journal.pone.0052892

**Published:** 2012-12-21

**Authors:** Jose Rafael Morillo Prado, Xin Chen, Margaret T. Fuller

**Affiliations:** 1 Department of Developmental Biology, School of Medicine, Stanford University, Stanford, California, United States of America; 2 Department of Biology, Johns Hopkins University, Baltimore, Maryland, United States of America; University of Bern, Switzerland

## Abstract

Adult stem cells are essential for the proper function of many tissues, yet the mechanisms that maintain the proper identity and regulate proliferative capacity in stem cell lineages are not well understood. Polycomb group (PcG) proteins are transcriptional repressors that have recently emerged as important regulators of stem cell maintenance and differentiation. Here we describe the role of Polycomb Repressive Complex 1 (PRC1) genes *Posterior sex combs* (*Psc*) and *Suppressor of zeste two* (*Su(z)2*) in restricting the proliferation and maintaining the identity of the Cyst Stem Cell (CySC) lineage in the *Drosophila* testis. In contrast, *Psc* and *Su(z)2* seem to be dispensable for both germline stem cell (GSC) maintenance and germ cell development. We show that loss of *Psc* and *Su(z)2* function in the CySC lineage results in the formation of aggregates of mutant cells that proliferate abnormally, and display abnormal somatic identity correlated with derepression of the Hox gene *Abdominal-B*. Furthermore, we show that tumorigenesis in the CySC lineage interferes non-cell autonomously with maintenance of GSCs most likely by displacing them from their niche.

## Introduction

Many adult tissues such as blood, skin, and the epithelial lining of the intestine and colon, require a constant supply of newly formed cells produced by the differentiated progeny of adult stem cells. The mechanisms that regulate and maintain cell identity and fate in adult stem cell lineages are thus crucial for long-term tissue maintenance and repair. Reciprocally, defects in the mechanisms that regulate stem cell self-renewal versus differentiation and maintain such fate decisions may contribute to tumorigenesis, as many human cancers arise in adult stem cell lineages [1].

Polycomb Group (PcG) proteins have been shown to play an important role in regulating cell fate and stem cell function, and their misregulation may lead to changes in the identity of a stem cell lineage or even cancer [2]. PcG proteins are conserved epigenetic regulators that act in multimeric complexes to maintain, through cell divisions, the spatially restricted expression of axial *Hox* genes set up during embryogenesis [3], [4], [5], as well as other targets. PcG proteins are organized in at least two different complexes: PRC1 and PRC2. PRC2 catalyzes methylation of lysine 27 of histone H3 and recruits the PRC1 complex. In *Drosophila*, PRC1 contains four core proteins that include Polycomb (Pc), Polyhomeotic (Ph), Sex combs extra (Sce/dRING), and Posterior sex combs (Psc) [6]. Two additional *Drosophila* homologues of Psc, encoded by the genes *Su(z)2* and *l(3)73Ah* [7], [8], [9] may participate in alternate forms of the PRC1 complex. In mammals, PRC1 members Mel18 and BMI-1, homologues of *Psc* and *Su(z)2*, play an important role in adult stem cell lineages. BMI-1 is required cell autonomously for self-renewal of neural and hematopoietic stem cells (HSC) [9], [10], [11], [12], while Mel18 acts as a tumor suppressor and controls the self-renewal and cell cycle of HSCs [13], [14], [15], [16], [17].

Here we use the *Drosophila* testis to investigate the role of BMI-1 and Mel18 homologues in maintaining cell fate and identity in adult stem cell lineages. The *Drosophila* testis is a powerful system for the study of adult stem cells [18], [19], [20]. Two adult stem cell populations reside at the apical tip of the *Drosophila* testes: germline stem cells (GSCs), which give rise to sperm, and cyst stem cells (CySCs), which give rise to the cyst cells that enclose and are required for the proper differentiation of germ cells [21], [22]. Both GSCs and CySCs are localized to the apical tip of the testis, attached to a cluster of postmitotic cells termed the hub.

In *Drosophila*, axial Hox gene *Abd-B*, a target of PcG proteins, plays an important role in gonad development during embryogenesis in males [23]. *Abd-B* is expressed in and required for the specification of posterior somatic gonadal precursors (SGPs) and it is absent from anterior SGPs, from which the hub and likely the CySCs derive [23], [24]. Conversely, ectopic *Abd-B* expression abolishes the specification of anterior SGPs [24]. Therefore, there must be a mechanism to maintain through development the repression of *Abd-B* established in anterior SGPs to allow for the proper specification of the hub and CySCs. PcG proteins might play such a role.

Here we present evidence that maintenance of the repressed state of *Abd-B* established in the anterior SGPSs during embryogenesis is important for the proper behavior and function of cells in the CySC lineage in adult testes. We show that *Psc* and *Su(z)2* act redundantly to maintain proper identity of the CySC lineage by repressing expression of *Abd-B*, while *Psc* and *Su(z)2* appear to be dispensable in the GSC lineage. In addition, we show that Psc and Su(z)2 act redundantly as tumor suppressors, and that tumorigenesis in the CySC lineage non-cell autonomously impairs maintenance of the germline by displacing neighboring GSCs from their niche.

## Materials and Methods

### Fly strains and husbandry

All fly stocks were raised on standard cornmeal/molasses or cornmeal/soy flour agar medium at 25°C unless stated otherwise. Strains are described in Flybase (http://flybase.org) and obtained from the Bloomington Stock Center unless specified otherwise. Flies used include the strains *yw;FRT42D, Psc^e24^/SM6b*, and *yw;FRT42D, Su(z)2^1.b7^/CyO*, both carrying loss of function PcG alleles, as well as *yw;FRT42D, lines^G2^/CyO* and *yw;FRT42D, Df(2R)Su(z)2^1.b8^/SM6b*, a deficiency removing both *Psc* and *Su(z)2*, as well as *Mdr49, CG3884, CG13321, CG33798, CG13323, CG13324,* and *Drl-2*, as indicated by PCR mapping. C587-GAL4; FRT42D, ubi-nGFP/CyO;tub-GAL80^ts^ or *nanos-GAL4; FRT42D, ubi-nGFP/CyO* flies were used for generating homozygous clones by FLP mediated mitotic recombination in the CySC and GSC lineages, respectively. *hs-FLP^122^; FRT42D, ubi-nGFP/CyO* flies were used for inducing clones ubiquitously by heat shock. *FRT42D* flies were used as control. Mutant and wild-type controls flies carrying a *UAS-FLP* transgene were used for tissue-specific clonal analysis. *C587-GAL4;tub-GAL80^ts^/CyO* flies were used in crosses to drive expression of transgenes including RNAi hairpins in the CySC lineage. *C587-GAL4;tub-GAL80^ts^/CyO* or *yw;Tj-GAL4* flies were used in crosses to drive ectopic expression of Abd-B in the CySC lineage under the control of UAS regulatory sequence. RNAi hairpin flies were obtained from either the Vienna *Drosophila* RNAi Center (VDRC) [25] or the Transgenic RNAi Project (TRiP) and included hairpins against *Psc* (FBst0458561) and *Su(z)2* (FBst0468996). Other fly strains included *w;UAS-Abd-B* (FBst0000913).

### Immunofluorescence

Testis samples were dissected in 1X PBS and fixed in 4% formaldehyde in phosphate-buffered saline (PBS) for 20 minutes at room temperature, washed twice for 30 minutes per wash in PBS containing 0.3% Triton X-100 and 0.6% sodium deoxycholate. Testes were incubated overnight at 4°C in primary antibodies. Primary antibodies included anti-Armadillo (Arm) (mouse, 1∶10) [26], anti-Eya (mouse, 1∶10) [27], anti-N-Cadherin (N-Cad) (rat, 1∶10) [28], and anti-E-cadherin (E-Cad) (rat, 1∶10) [29] obtained from the Developmental Studies Hybridoma Bank. Other antibodies included anti-Abdominal-B (mouse, 1∶10) [30], [31]; anti-Green Fluorescent Protein (GFP) (rabbit, 1∶500–1∶1000; Invitrogen), anti-phospho Histone H3 Thr3 (rabbit, 1∶100) (Millipore), anti-Traffic-Jam (guinea pig 1∶1000) (gift from Dr. Dorothea Godt) [32], anti-Ultrabithorax (mouse, 1∶10) (gift from Dr. Rob White and Dr. Richard Cripps) [33], anti-Vasa (goat, 1∶50−1∶100) (Santa Cruz Biotechnology, Inc), and anti-Zinc finger homeodomain-1 (Zfh-1) (rabbit, 1∶5000) (gift from Dr. Ruth Lehman). Antibodies against Psc and Su(z)2 used successfully in other tissues, including those from the DSHB [34], yielded non-specific staining in testes. Staining of wild-type testes showed what appeared to be chromatin and perinuclear staining. However, such staining was also present in *Df(2R)Su(z)2^1.b8^* homozygous null mutant clones, which lack both *Psc* and *Su(z)2*. Several fixation methods were also tested unsuccessfully including the use of methanol or formaldehyde instead of paraformaldehyde (not shown). Secondary antibodies were from the Alexa Fluor-conjugated series (1∶200; Molecular Probes). Chromatin was visualized by DAPI (4′,6-diamidino-2-phenylindole) staining. Samples were mounted on VECTASHIELD medium (Vector Labs H-1200). Immunofluorescence images were obtained with a Leica SP2 AOBS Confocal Laser Scanning microscope. Phase images were obtained using a Zeiss Axioskop microscope and a SPOT RT3 camera by Diagnostic Instruments, Inc. Clonal analysis images were obtained using a Zeiss Axioplan microscope and a CoolSNAPez camera by Photometrics or a Samsung SGH E315. Images were processed using Adobe Photoshop CS4 and Adobe Illustrator CS4 software.

### Clonal and RNAi Analysis

Homozygous mutant clones were induced in a heterozygous background by expressing FLP recombinase either ubiquitously for a short, defined period by heat shock [35] or in a tissue specific manner. CySC and GSC lineage clones were generated by using females carrying the *C587-GAL4* or the *nos-GAL4* drivers, respectively, in combination with a *UAS-FLP* transgene. We found that while the C587-GAL4 driver works primarily in the CySC lineage, it might also drive expression of transgenes in the germline at low levels based on the appearance of some germ cell clones (Fig. S1). Ubiquitous clones were generated by using females carrying a *hs-FLP^122^* transgene. Virgin females carrying hs-FLP were crossed to *w;FRT42D* or *yw;FRT42D, Psc^e24^/CyO* or *yw;FRT42D, Su(z)2^1.b7^/CyO* or *Df(2R)Su(z)2^1.b8^/SM6b* males. For heat-shock induced clones, flies were grown at 25°C and heat-shocked at 37°C for two hours on each of two consecutive days at the pupal stage. The size of resulting aggregates of mutant cells was obtained by quantifying the area of nGFP negative mutant aggregates in squashed preparations in pixels (Fig. S3). This method likely underestimates the increase in volume of the aggregates, but since the preparations were squashed it is not possible to use a simple calculation to estimate the volume from the area, so we chose to plot the more conservative measure of area in images. For CySC and GSC lineage specific clones, flies carrying a *tub-GAL80^ts^* transgene and either C587-GAL4 or Nanos-GAL4 were raised at 18°C until eclosion and transferred to 30°C for clone induction. The resulting homozygous mutant cells were negatively marked and identified by their lack of nGFP and with antibodies against the GFP protein.

RNAi knockdown experiments were carried out by crossing flies carrying RNAi hairpins under the UAS regulatory sequence to *C587-GAL4;tub-Gal80^ts^/CyO* or *Sco/CyO;NG4VP16* females. The progeny were raised at 18°C until eclosion, then transferred to and held at 30°C to induce expression. Expression of *Psc* and *Su(z)2* in the CySC or GSC lineages after tissue specific RNAi could not be assessed by qRT-PCR to document knockdown, as experiments were performed in whole testes, which include both germ cells and several different types of somatic cells. As a result, decrease in abundance of the *Psc* and *Su(z)2* mRNAs after RNAi would be obscured by the presence in the same testis of abundant cell types in which RNAi was not expressed. However, knockdown of *Psc* and *Su(z)2* by RNAi in the somatic cyst cell lineage clearly had an effect, because it cause a phenotype, notably ectopic expression of *Abd-B*, also seen for testis somatic cells made homozygous mutant for a deletion of both *Psc* and *Su(z)2*.

## Results

### *Psc* and *Su(z)2* are functionally redundant and required cell autonomously in the CySC but not the GSC lineage

**Figure 1:**
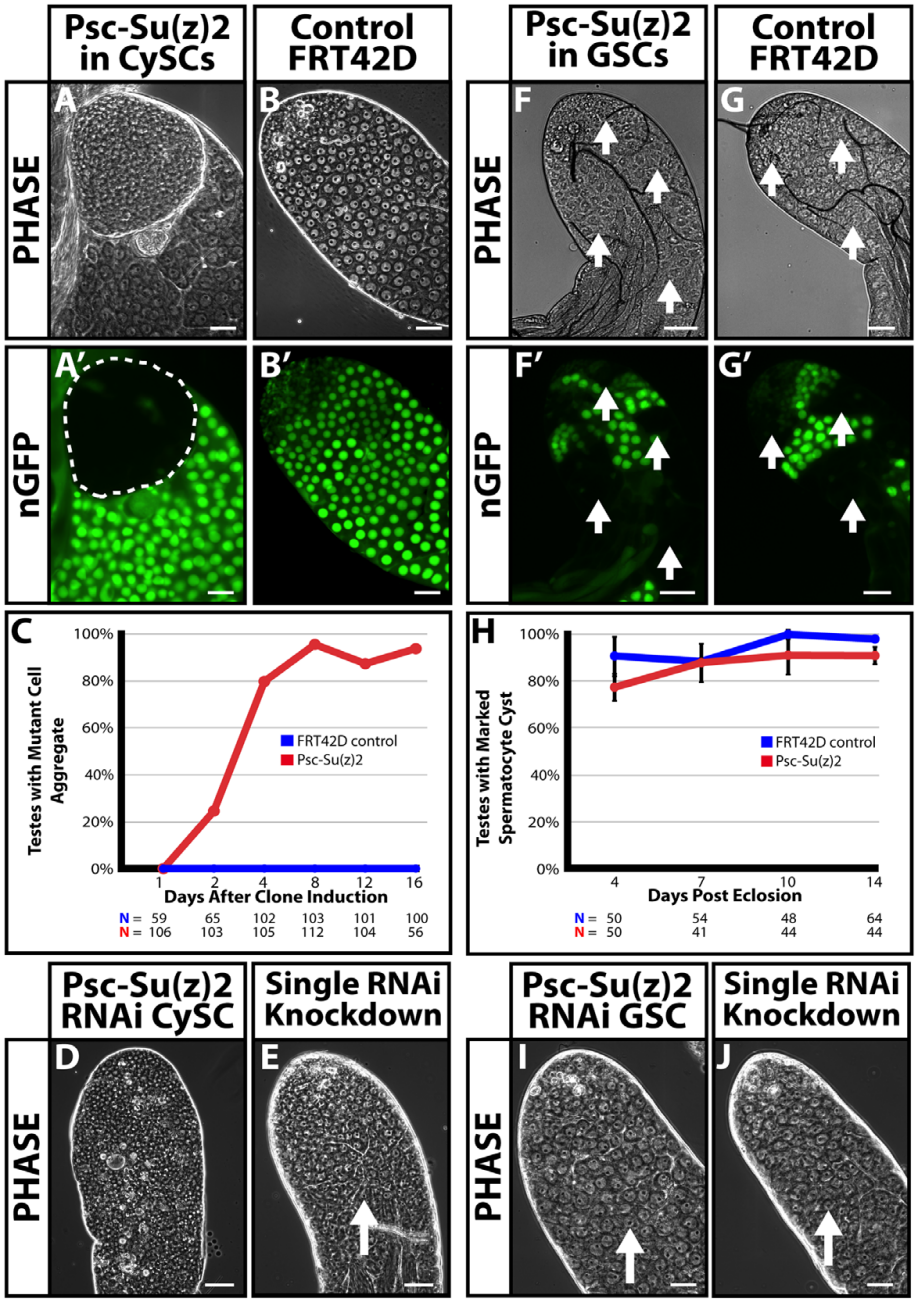
*Psc* and *Su(z)2* are redundant and required in the CySC but not the GSC lineage. (**A–C**): Analysis of testes with *Psc* and *Su(z)2* double mutant clones generated in the CySC lineage (*C587-GAL4;FRT42D, Df(2R)Su(z)2^1.b8^/FRT42D, ubi-nGFP;UAS-FLP/tub-GAL80^ts^*) and control (*C587-GAL4;FRT42D/FRT42D, ubi-nGFP;UAS-FLP/tub-GAL80^ts^*). (A–A') Marked nGFP-negative *Psc* and *Su(z)2* double mutant cell aggregate (dashed line). (B–B') Control clones of normal cyst cells homozygous for FRT42D. (A, B) Phase; (A', B') nGFP. Testes examined 8 days after clone induction. (**C**): Percentage of testes with a visible abnormal cell aggregate at the tip of the testis scored at different time points after induction of *Psc* and *Su(z)2* double mutant clones in the CySC lineage. (Red line) Testes with *Psc* and *Su(z)2* double mutant clones. (Blue line) FRT42D controls. (**D–E**): Phase-contrast images of apical tip of testes with (D) simultaneous RNAi knockdown of *Psc* and *Su(z)2* (*C587-GAL4;UAS-Psc-RNAi/UAS-Su(z)2-RNAi;tub-GAL80^ts^*) or (E) single RNAi knockdown of either *Psc* (*C587-GAL4;UAS-Psc-RNAi/CyO;tub-GAL80^ts^*) or *Su(z)2* (*C587-GAL4;UAS-Su(z)2-RNAi/CyO;tub-GAL80^ts^*) alone in the CySC lineage (phenotypically indistinguishable). Testes 12 days after RNAi induction. Arrow marks spermatocytes. (**F–H**): Analysis of testes with *Psc* and *Su(z)2* double mutant clones generated in the GSC lineage. (F–F') *Psc* and *Su(z)2* double mutant clones homozygous mutant for *Df(2R)Su(z)2^1.b8^* deficiency. (G–G') Control germline clones homozygous for FRT42D. Bright field (F, G); nGFP (F', G'). Arrows mark germline clones. Testes 7­ days after clone induction. (**H**): Percentage of testes with *Psc* and *Su(z)2* double mutant (red line) or wild-type control (blue line) spermatocyte cysts scored at different time points after eclosion. Data reported as average +/− S.D. (**I–J**): Phase images of apical tip of testes with RNAi knockdown in the GSC lineage of (I) both *Psc* and *Su(z)2* or (J) either *Psc* or *Su(z)2* controls (phenotypically indistinguishable). Testes 12 days after RNAi induction. Spermatocytes marked by arrows. Scale bars: 50 μm.

Loss of both *Psc* and *Su(z)2* function in the CySC lineage resulted in formation of an abnormal aggregate of mutant cells at the tip of the testis (Fig. 1A–A', dotted line) not seen in wild-type controls (Fig. 1B–B'). Negatively marked clones of cells homozygous for a deficiency deleting both *Psc* and *Su(z)2* were generated in the CySC lineage using the C587-GAL4 driver to express FLP recombinase. Temporal control of clone induction was provided by tub-GAL80^ts^ and flies were grown at 18°C and later shifted to 30°C within 24 hours of eclosion to induce mitotic recombination in the CySC lineage [36], [37]. By day 2 after clone induction, 24% of testes had one or more small abnormal aggregates of marked mutant cells at the apical tip of the testis. By day 16, over 90% of all testes contained a large abnormal aggregate of mutant cells (Fig. 1A–C). Consistent with the clonal analysis, and indicating that the effects observed were due to loss of function of both *Psc* and *Su(z)2* rather than other loci removed by *Df(2R)Su(z)2^1.b8^*, simultaneous knockdown of both *Psc* and *Su(z)2* in the CySC lineage using RNAi hairpins expressed under the control of the C587-GAL4 driver resulted in testes with an abundance of small cells by day 12 (Fig. 1D). Testes from siblings with conditional knockdown of either *Psc* or *Su(z)2* alone appeared normal by phase-contrast microscopy (Fig. 1E), indicating that *Psc* and *Su(z)2* are genetically redundant in the CySC lineage. Notably, knockdown of *Psc* and *Su(z)2* in the CySC lineage resulted in testes with few or no spermatocytes by day 12 (Fig. 1D) after RNAi induction, indicating that the state of somatic cells influences germ cell differentiation.

In contrast to their role in the CySC lineage, *Psc* and *Su(z)2* were not required cell autonomously for GSC maintenance or germ cell differentiation. Generation of marked clones of cells homozygous mutant for *Df(2R)Su(z)2^1.b8^*exclusively in the GSC lineage using the nanos-GAL4 driver did not result in formation of abnormal aggregates of mutant cells at the tip of the testis (Fig. 1F–G'). In addition, marked spermatocyte cyst clones lacking both *Psc* and *Su(z)2* function (*Df(2R)Su(z)2^1.b8^*/*Df(2R)Su(z)2^1.b8^*; Fig. 1F–F', arrows) were maintained at the same rate as wild-type controls clones (Fig. 1G–G', arrows) for at least 14 days after clone induction, suggesting that mutant GSCs remained functional and able to produce differentiating progeny (Fig. 1H). Strikingly, mutant germ cells were able to differentiate normally through the spermatogonial, spermatocyte, and spermatid stages (Fig. S2A–C', dotted line). Similar results were observed when germline clones lacking either *Psc* or *Su(z)2* function alone were generated (Fig. S2D–G). Likewise, simultaneous knockdown in the germline of both *Psc* and *Su(z)2* or either *Psc* or *Su(z)2* alone did not impair spermatocyte differentiation (Fig. 1I-J, arrows).

### Cells in *Psc* and *Su(z)2* double mutant aggregates are mitotically active

**Figure 2:**
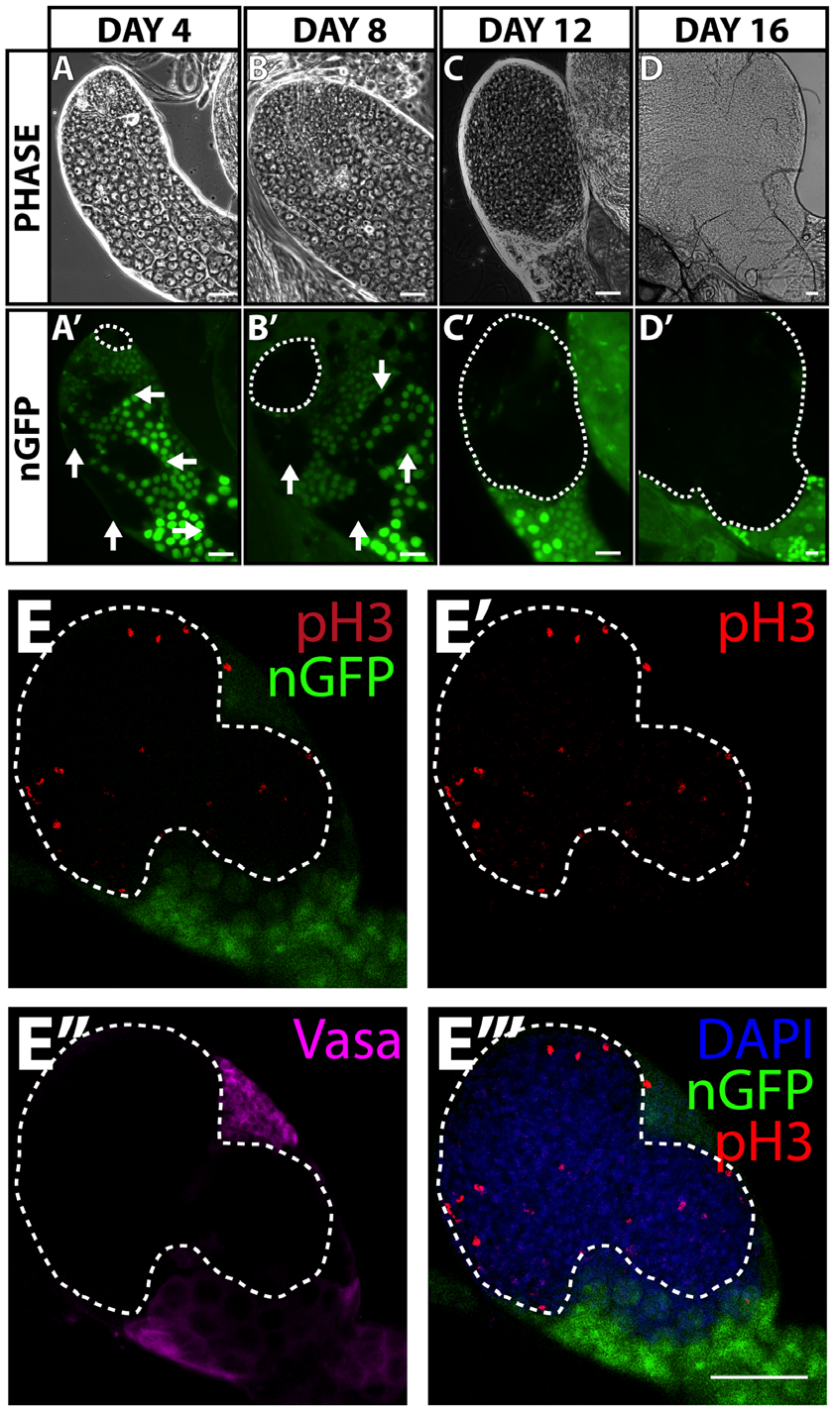
Cells in *Psc* and *Su(z)2* double mutant aggregates are mitotically active. (**A–D**) Phase-contrast and bright field images of apical region of testes with *Psc* and *Su(z)2* double mutant clones generated by heat shock FLP and marked by the absence of nGFP (*hs-FLP;FRT42D, Df(2R)Su(z)2^1.b8^/FRT42D, ubi-nGFP*) (A, A') Day 4, (B, B') day 8, (C, C') day 12, and (D, D') day 16 after clone induction. (Dashed line) Abnormal *Psc* and *Su(z)2* mutant cell aggregates. (Arrows) Germline clones. Phase (A, B, C), nGFP (A', B', C', D'). Bright field (D). (**E–E’’’**): Confocal microscopy projection through a testis containing a *Psc* and *Su(z)2* mutant aggregate 16 days after clone induction immunostained for (E–E’’’) phospho-Histone H3 (pH3) to mark mitotically active cells, (E’’) Vasa to mark germ cells, and (E’’’) DAPI to mark DNA. (E) merged image of E'-E’’’. Scale bars: 50 μm.

The mutant cell aggregates lacking *Psc* and *Su(z)2* that accumulated at the tip of the testis increased in size over time (Fig. 2A–D', Fig. S3), and were composed of mitotically active cells. When cells homozygous mutant for *Psc* and *Su(z)2* were induced by heat shock, the average size of mutant aggregates increased over time as measured by area in pixels (Fig. S3). The increase in size was not due to the generation and accumulation of new mutant cell aggregates, as mutant clones were generated by two short pulses of FLP expression under heat shock control on two consecutive days only at the pupal stage. In addition, and consistent with continued proliferation of *Psc* and *Su(z)2* double mutant cells, cells throughout the abnormal mutant aggregate and well away from the apical hub stained positive for the mitotic marker phospho-histone H3 (Fig. 2E–E’’’), unlike CySCs which do so exclusively close to the hub. The abnormal aggregates of mutant cells did not express the germline marker Vasa (Fig. 2E), consistent with the requirement for *Psc* and *Su(z)2* in the CySC lineage but not the germline documented in Figure 1.

### Loss of *Psc* and *Su(z)2* function in the CySC lineage impaired GSC function non-cell autonomously

**Figure 3:**
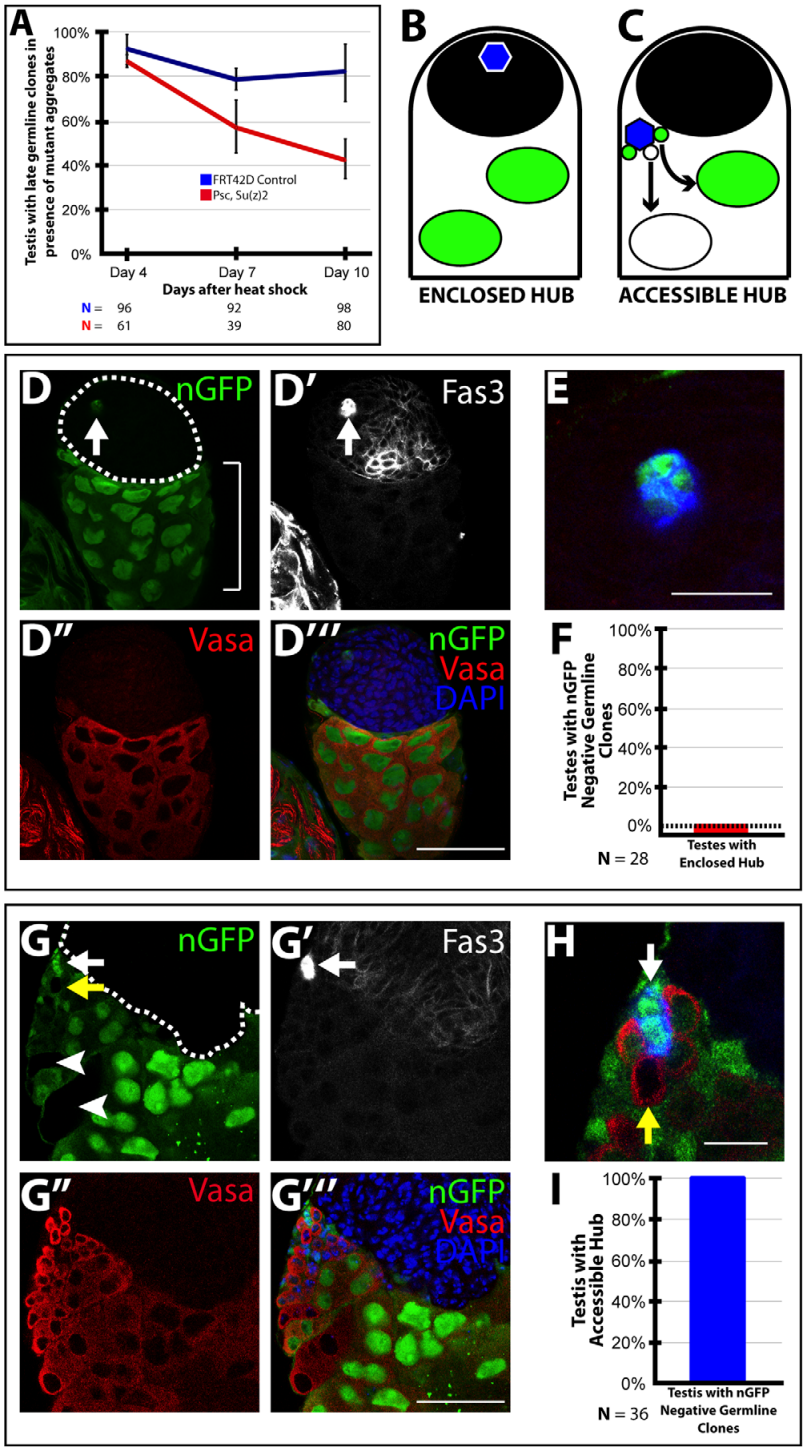
Loss of *Psc* and *Su(z)2* function in the CySC lineage impairs GSC maintenance non-cell autonomously. (**A**): Maintenance of marked nGFP-negative late germline clones (spermatocyte and spermatid) in testes with (red line) *Psc* and *Su(z)2* double mutant aggregates (*hs-FLP;FRT42D, Df(2R)Su(z)2^1.b8^/FRT42D, ubi-nGFP*) or (blue line) FRT42D control (*hs-FLP;FRT42D/FRT42D, ubi-nGFP*) clones generated randomly in the GSC and CySC lineages by heat shock and scored at different times after clone induction. Data reported as average +/− S.D. (**B–C**): Schematic representation of testes containing a hub (blue) (B) enclosed by the mutant cell aggregate or (C) displaced to the side of a mutant cell aggregate and accessible to GSCs. (White) nGFP negative GSC clone. (Black) abnormal mutant cell aggregate. (**D–E**): Apical tip of a testis 8 days after clone induction showing hub enclosed by mutant cell aggregate (enclosed hub). Testis stained with antibodies against (D, D’’’) nGFP, arrow shows enclosed hub lacking GSCs; (D') Fas3 to mark the hub, (D’’, D’’’) Vasa to mark germ cells, and (D’’’) DAPI to mark DNA. (E) Close up of hub taken from merged images D–D’’ with Fas3 in blue. White arrow marks the hub; bracket shows presence of spermatocytes and lack of early germ cells. (**F**): Quantification of marked nGFP negative germline clones in testes with enclosed hub 15 days after clone induction. (**G–H**): Apical tip of a testis 8 days after clone induction showing a hub outside the mutant cell aggregate and associated with neighboring GSCs (accessible hub). (G, D’’’) Testis stained with antibodies against nGFP, (G', white arrow) Fas3 to mark the hub, (G’’, D’’’) Vasa to mark germ cells, and (G’’’) DAPI to mark DNA. (H) Merged close up of hub taken from images G–G’’ with Fas3 in blue. White arrow marks the hub, yellow arrow marks nGFP negative GSC, arrowheads mark germline clones. (**I**): Quantification of accessible hubs in testes with marked nGFP germline clones 15 days after clone induction. Scale bars 50 µm except in E and H where scale bar is 20 µm.

Although *Psc* and *Su(z)2* were not required cell-autonomously in the germline for stem cell maintenance, the formation of aggregates of proliferating cells double mutant for *Psc* and *Su(z)2* derived from the CySC lineage interfered with GSC maintenance non-cell autonomously, most likely because the aggregate in some cases surrounded the hub, displacing GSCs from their niche. When clones of cells double mutant for *Psc* and *Su(z)2* were induced at random in both the germ line and somatic cell lineages by heat shock, analysis of those testes in which a mutant cell aggregate was formed revealed that, in a subset of cases, the negatively marked germline clones induced in the same testis were often not maintained (Fig. 3). By day 10 after induction of clones double mutant for *Psc* and *Su(z)2* in the pupal stage, only 42% of testes examined had marked germline clones, compared to 82% of testes in which wild-type control clones were induced, in which no aggregate of mutant somatic cells arose (Fig. 3A, compare to figure 1H).

Further analysis of the special relationship between the aggregates of mutant somatic cells and the apical hub revealed an explanation for why some testes lacked germline clones while others maintained clones of marked germ cells. Abnormal aggregates of proliferating cells derived from the CySC lineage appeared to deplete the germline by displacing GSCs from their normal supportive niche. By day 15 after clone induction at the pupal stage, testes containing mutant cell aggregates had two distinct spatial arrangements of the hub with respect to the abnormal aggregate of mutant cells (Fig. 3B, C). Those testes that contained hubs that remained at the apical tip but were enclosed by the mutant cell aggregate (enclosed hubs, Fig. 3B) lacked both wild-type and mutant GSCs altogether (Fig. 3D–E) and also lacked marked germline clones by day 15 after clone induction (Fig. 3F). Many of these testes also contained only late germ cells such as spermatocytes and spermatids but no early germ cells such as spermatogonia (Fig. 3D, bracket), indicating prior loss of wild-type as well as mutant GSCs. Conversely, those testes that contained hubs outside of the mutant cell aggregate, commonly displaced from the apical tip of the testis (accessible hubs, Fig. 3C), were associated with both wild-type and mutant GSCs (Fig. 3G-H; white arrow, hub; yellow arrow, marked GSC; arrowhead, marked germline clones). Moreover, 100% of all testes observed after day 15 post-clone induction that had a mutant cell aggregate and marked germline clones contained accessible hubs associated with marked GSCs (Fig. 3I).

### Cells in the mutant aggregates exhibit abnormal somatic cell identity

**Figure 4:**
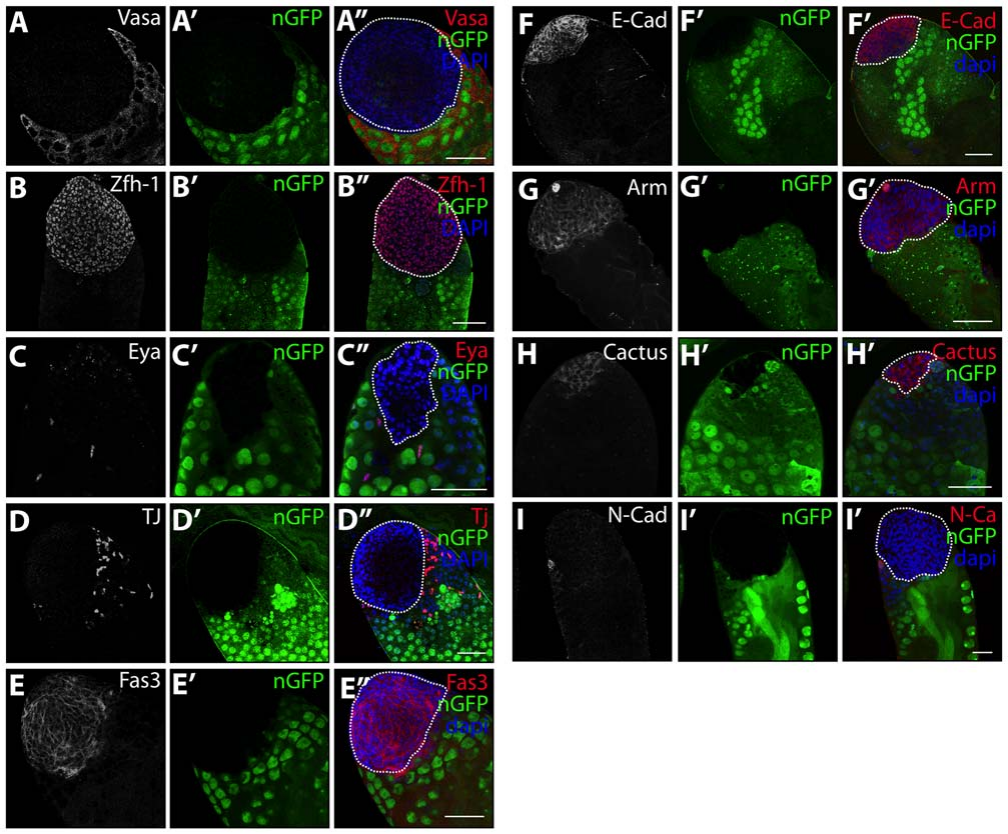
Cells in mutant aggregates express some markers of somatic cells of the testis. (**A–I'**): Immunostain of testes with abnormal *Psc* and *Su(z)2* double mutant cell aggregates 8 days after clone induction (*hs-FLP;FRT42D, Df(2R)Su(z)2^1.b8^/FRT42D, ubi-nGFP*). Testis immunostained with DAPI (blue), and the following antibodies: anti-GFP (green), (A–A’’) anti-Vasa, (B–B’’) anti-Zfh-1, (C–C’’) anti-Eya, (D–D') anti-Tj, (E–E’’) anti-Fas3, (F–F’’) anti-E-Cad, (G–G’’) anti-Arm, (H–H’’) anti-Cactus, and (I–I’’) anti-N-Cad. Stain of control testes with FRT42D clones shown in Fig. S4. Scale bars: 50 μm.

Consistent with the requirement for *Psc* and *Su(z)2* in the CySC but not the GSC lineage (Fig. 1), the abnormal aggregates of proliferating cells lacked expression of the germline marker Vasa (Fig. 2E’’; 3D’’, G’’; Fig. 4A–A’’). However, immunostaining revealed that the *Psc* and *Su(z)2* double mutant cells in the aggregates had abnormal somatic cell identity (compare to immunostained control testes in Figure S4) . Mutant cells expressed high levels of Zfh-1 (Fig. 4B–B’’), a marker commonly associated with CySCs and early cyst cells (Fig. S4B–B') [38], and lacked detectable levels of Eya (Fig. 4C–C’’), a marker of differentiated cyst cells and the terminal epithelium (Fig. S4C–C') [39], [40]. Surprisingly, the cells in the mutant aggregates were negative for Tj (Fig. 4D–D’’), a marker of the CySC lineage that is also expressed in the hub at low levels (Fig. S4D–D') [32]. Cell aggregates lacking *Psc* and *Su(z)2* stained positive for Fas3 (Fig. 4E–E’’) and Arm (Fig. 4G–G’’), both expressed in the hub and the terminal epithelium, as well as for E-Cad (Fig. 4G–G’’) and Cactus (Fig. 4H–H’’), which are also expressed in the hub (Fig. S4E–H'). However, the cell adhesion molecule N-Cad, which is normally expressed in hub cells but not CySC or cyst cells (Fig. S4I–I'), was not detected in the mutant aggregates (Fig. 4I–I’’). These data suggest that loss of *Psc* and *Su(z)2* caused cells of the CySC lineage to take on an abnormal somatic identity.

### *Psc* and *Su(z)2* are required in the CySC but not the GSC lineage to maintain repression of *Abdominal-B*

**Figure 5:**
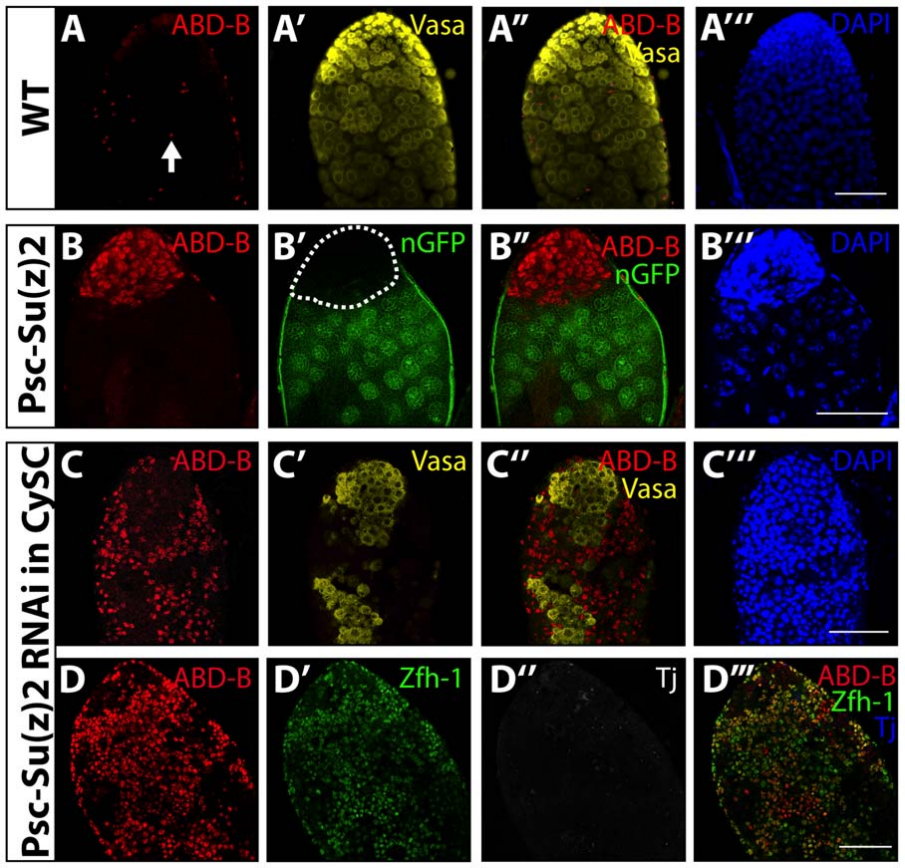
*Psc* and *Su(z)2* are required in the CySC lineage for repression of *Abd-B.* (**A–B’’’**): Immunofluorescence images of testes from (A–A’’’) newly eclosed males with wild-type control or (B–B’’’) *Psc* and *Su(z)2* double mutant clones generated in the CySC lineage (*C587-GAL4;FRT42D, Df(2R)Su(z)2^1.b8^/FRT42D, ubi-nGFP;UAS-FLP/tub-GAL80^ts^)*, immunostained with anti-ABD-B (red), anti-Vasa (A', A’’, yellow), anti-GFP (B', B’’, green), and DAPI (blue). Arrow marks nucleus of a sheath cell. (**C–D’’’**): Testes with simultaneous RNAi knockdown of both *Psc* and *Su(z)2* in the CySC lineage (*C587-GAL4;UAS-Psc-RNAi/UAS-Su(z)2-RNAi;tub-GAL80^ts^*) immunostained with anti-ABD-B (red), anti-Vasa (C', C’’, yellow), anti-Zfh-1 (D', D’’’ green), anti-Tj (D’’, grey), and DAPI (C’’’, blue). Scale bars: 50 μm.

Loss of *Psc* and *Su(z)2* function in the CySC lineage resulted in derepression of the *Hox* gene *Abdominal-B (Abd-B)*. Immunostaining of wild-type testes revealed ABD-B protein in sheath cell nuclei (Fig. 5A, arrow), but not in either the GSC or the CySC lineages. In contrast, immunostaining revealed expression of ABD-B in cells of the mutant aggregates lacking Psc and Su(z)2 (Fig. 5B–B’’). Furthermore, simultaneous knockdown of both *Psc* and *Su(z)2* function in the CySC lineage by RNAi resulted in derepression of ABD-B (Fig. 5C–D’’’). The mass of somatic cells that filled the testes after conditional knockdown of *Psc* and *Su(z)2* by RNAi in the CySC lineage expressed Zfh-1 (Fig. 5D') and lacked Tj (Fig. 5D’’) and Vasa (Fig. 5C', C’’), indicating that the defects observed in the clonal analysis were due to the loss of *Psc* and *Su(z)2* function, not of other loci removed by the *Df(2R)Su(z)2^1.b8^* deletion. Testes from control flies in which UAS-GFP was driven under the control of C587-GAL4 appeared normal by phase-contrast microscopy (not shown).

The CySC and GSC lineages differed in their requirement for *Psc* and *Su(z)2* to repress *Abd-B*. Germ cell clones lacking *Psc* and *Su(z)2* function lacked detectable ABD-B (Fig. S5A', yellow dashed lines) in GSCs (Fig. S5B–B'), spermatogonia (Fig. S5C–C'), and spermatocytes (Fig. S5D–D'). Consistent with the clonal analysis, no ABD-B was detected after simultaneous knockdown of *Psc* and *Su(z)2* function by RNAi in the GSC lineage (Fig. S5E, compare to figure 5C ).

### Ectopic expression of *Abdominal-B* in the CySC lineage partially phenocopied the loss of *Psc* and *Su(z)2*

**Figure 6:**
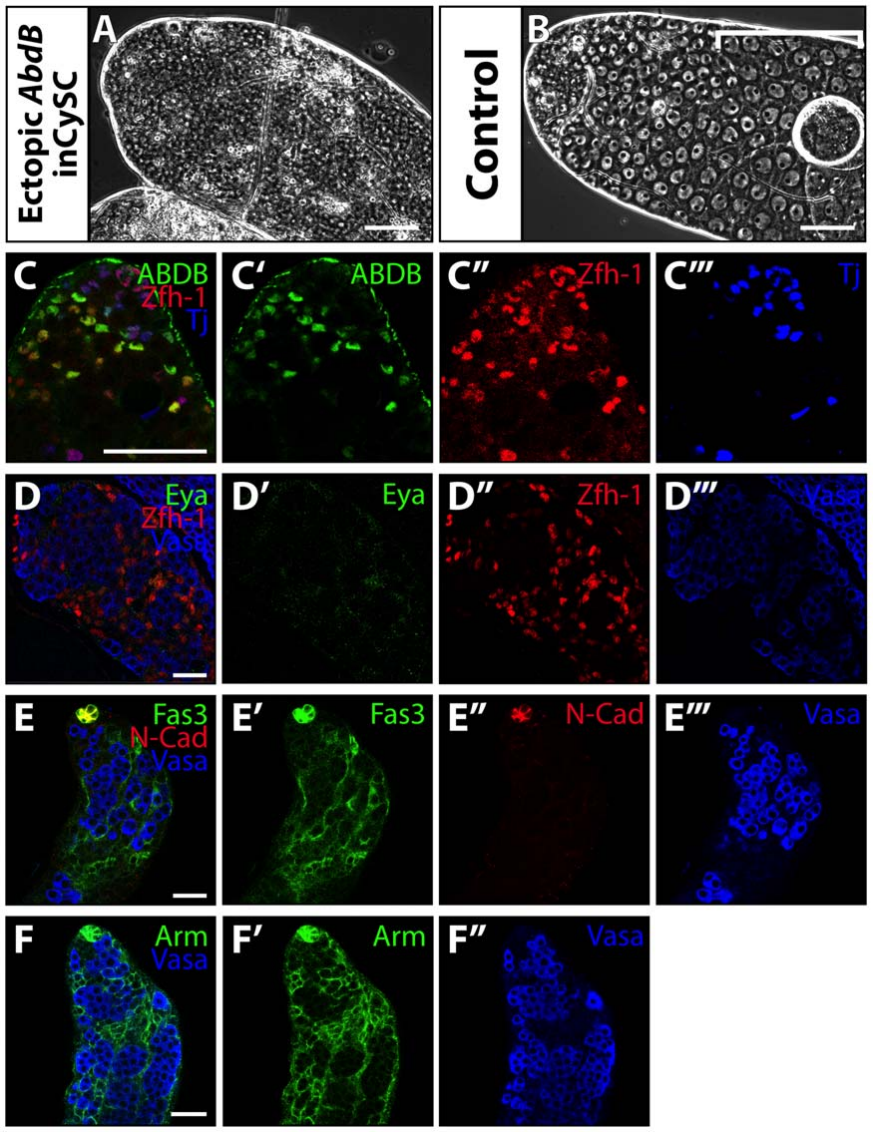
Ectopic expression of *Abdominal-B* in the CySC lineage partially phenocopies loss of *Psc* and *Su(z)2.* (**A–B**): Phase images of apical tip of testis 12 days after (A;C–F’’’) forced ectopic expression of *Abd-B* in the CySC lineage (*C587-GAL4;UAS-Abd-B;tub-GAL80^ts^*) and (B) control (*UAS-Abd-B;tub-GAL80^ts^*). Spermatocytes marked by bracket. (**C–F’’’**): Testes immunostained with (C, C') anti-ABDB, (C, C’’, D, D’’) anti-Zfh-1, (C, C’’’) anti-Tj, (D, D') anti-Eya, (D, D’’’, E, E’’’, F, F’’’) anti-Vasa, (D, D’’’, E, E’’’, F, F’’,) anti-Fas3, (E, E’’) anti-N-Cad, and (F, F') anti-Arm, antibodies. Scale bars: 50 µm.

Forced expression of *Abd-B* in the CySC lineage by using C587-GAL4 to drive the *Abd-B* cDNA under the UAS regulatory sequence resulted in testes with an abundance of small cells (Fig. 6A–B), reminiscent of the phenotype observed upon RNAi knockdown of both *Psc* and *Su(z)2* in the CySC lineage (compare to Fig. 1D). Like the mutant somatic cell aggregates lacking *Psc* and *Su(z)2*, these testes had an abundance of somatic cells with abnormal identity. Immunostaining of testes with forced expression of *Abd-B* in the CySC lineage revealed 49% of testes had somatic cells expressing detectable ABD-B protein (Fig. 6C–C') and expanded expression of Zfh-1 (Fig. 6C, C’’), which is normally restricted to CySCs and early cyst cells (n = 35). However, 90% of testes also had detectable levels of Tj protein (n = 35). Staining with antibodies against ABD-B, Zfh-1 and Tj revealed that 96% of Abd-B expressing cells also expressed Zfh-1, but only 13% expressed Tj (n = 55), consistent with the profile of cells lacking Psc and Su(z)2 function (Fig. 4). The levels of ABD-B detected varied from cell to cell, and not all cells had detectable ABD-B (Fig. 6). The presence of Tj positive cells may reflect insufficient expression of *Abd-B* in some cells. Unfortunately, due to antibody incompatibility, we were unable to determine to what extent ABD-B expressing cells also expressed Eya. However, Eya was detected at low levels in 23% of testes and was absent in 77% of testes (n = 26) (Fig. 6D, D'). As observed in *Psc* and *Su(z)2* mutant clones, ectopic expression of Abd-B also resulted in expanded expression of cell adhesion molecules Fas3 (Fig. 6E–E') and Arm (Fig. 6F–F') but not of N-Cad (Fig. 6E, E’’). Finally, ectopic expression of Abd-B in the CySC lineage resulted in lack of differentiation of the germline, as indicated by the lack of spermatocytes (Fig. 6A–B, bracket).

### *Psc* and *Su(z)2* double mutant cell aggregates behaved similarly but not identically to *lines* mutant clones

Previous studies found that the gene *lines* is also required to maintain the identity of the CySC lineage [41]. Although cells in the aggregates derived from the testis CySC lineage lacking *Psc* and *Su(z)2* displayed several characteristics similar to *lines* mutant clones, they differed in their effects on *Abd-B*, *N-Cad*, and cell proliferation. As for *Psc* and *Su(z)2* double mutant aggregates, clones of somatic cells mutant for *lines^G2^* expressed *Zfh-1* (Fig. S6A') but lacked Eya (Fig. S6A’’) and Tj (Fig. S6B'). Similar to hub cells, clones of somatic cells mutant for *lines* also expressed high levels of E-Cad (Fig. S6B’’), Arm (Fig. S6C'), Fas3 (Fig. S6D'), and Cactus [41], as did the aggregates of somatic cells double mutant for *Psc* and *Su(z)2* (Fig. 4). However, unlike the *Psc* and *Su(z)2* double mutant aggregates, *lines* mutant clones expressed *N-Cad* (Fig. S6D’’) and lacked detectable levels of ABD-B (Fig. S6E'). Most notably, although *lines^G2^* mutant clones initially proliferated, they eventually withdrew from the cell cycle [41], while somatic cells lacking *Psc* and *Su(z)2* continued to proliferate (Fig. 2).

## Discussion

The PcG proteins Psc and Su(z)2 act redundantly and cell-autonomously in the CySC lineage in *Drosophila* testes where they serve as tumor suppressors, repress the Hox gene *Abd-B*, and maintain the proper identity of the CySC lineage. The role of Psc and Su(z)2 in adult testes uncovered in this study may reflect a continued requirement for Psc and Su(z)2 function for maintenance of the proper domain of expression of Hox genes set up in somatic cells of the developing gonad during embryogenesis.

The development of the *Drosophila* male gonad involves the interaction and coordinated specification of embryonic germ cells and somatic gonadal precursors (SGPs), from which the adult germline and somatic cells of the testis originate. ABD-B is required in the embryonic gonad for specification of the posterior SGPs, including the male-specific SGPS (msSGPs). Conversely, *Abd-B* must be kept off in anterior SGPs for the proper specification of the CySC lineage [23], [24], [42]. PcG function is required in the embryonic gonad to provide this repression: Pc mutant embryos show misexpression of *Abd-B* in somatic cells throughout the embryonic gonad, resulting in lack of specification of the anterior SGPs that give rise to the hub and CySCs [23], [24], [42]. Our data indicate that Psc and Su(z)2 are also required during later development for continued repression of ABD-B and to maintain the identity of the CySC lineage in adult testes.

### Loss of Psc and Su(z)2 function and derepression of *Abd-B* are responsible for the abnormal identity of cells in mutant aggregates

The tumorigenic cells that arise upon loss of function of *Psc* and *Su(z)2* in the CySC lineage appear to have an abnormal identity, as they share some but not all markers of known somatic cell types in the adult testis. The high expression of Zfh-1 and lack of Eya in the mutant cells is reminiscent of CySCs and early cyst cells [38]. However, unlike either CySCs or hub cells, the *Psc* and *Su(z)2* mutant cells derived from the CySC lineage in the testis lacked Tj. Similarly, the mutant cells expressed a number of hub cell markers including Fas3, E-Cad, and Arm. Yet, they did not express the hub marker N-Cad. Although the expression of ABD-B in the *Psc* and *Su(z)2* double mutant somatic cells is reminiscent of msSGPs, and ABD-B is necessary and sufficient for specification of mSGPs during embryogenesis [23]. However, we did not detect SOX100B (data not shown) or Eya, both of which are expressed in msSGPs, in the *Psc* and *Su(z)2* double mutant cells. The mutant cells also resembled cells of the terminal epithelium, which derive from msSGPs [40], [43] and express *Fas3* and *Arm* but lack Tj. However, unlike the terminal epithelium, the mutant cells lacked Eya and expressed *Abd-B*, which we detected in sheath cells and the accessory glands.

Much of the abnormal identity of the *Psc* and *Su(z)2* double mutant somatic cells could be explained by the expression of *Abd-B* and lack of Tj. The phenotype observed upon loss of *Psc* and *Su(z)2* function in the CySC lineage was remarkably similar to that observed upon forced ectopic expression of *Abd-B* (compare Figs. 4, 5, and 6). In both cases cells became enriched for Zfh-1, Fas3, and Arm while they lacked Tj, Eya, and N-Cad. Previous work in the embryo indicates that ectopic expression of *Abd-B* is sufficient to change the identity of cells, resulting in the expression of various cell adhesion molecules including *E-Cad* [44]. One possibility might be that loss of *Psc* and *Su(z)2* function causes failure of somatic cells to express Tj because ABD-B represses expression of a gene required for expression of *Tj*. In *Drosophila* ovaries, follicle cells lacking Tj overexpress *Fas3* and *E-Cad*, while they show no change in N-Cad expression [32].

### *Psc*, *Su(z)2* and proliferation

Forced ectopic expression of *Abd-B* under our experimental conditions did not, however, phenocopy all aspects of the *Psc Su(z)2* mutant phenotype. The most striking phenotypic difference was in cell proliferation, suggesting that Psc and Su(z)2 may control the cell cycle independently of ABD-B. Loss of *Psc* and *Su(z)2* function in the CySC lineage resulted in continuous proliferation of the mutant cells, forming a large aggregate (Fig. 2). In contrast, forced expression of *Abd-B* in the CySC lineage resulted in only limited proliferation, as evident by the regular size of the testes (Fig. 6). PcG proteins are known to be involved in cell cycle regulation. For example, previous studies indicate that, in the *Drosophila* female gonad, loss of *Psc* and *Su(z)2* function results in the continuous self-renewal of follicle stem cells [45]. In *Drosophila*, the *CycA* locus contains a Polycomb response element (PRE) site that recruits the PRC1 complex [46], indicating direct control. Loss of *Pc* function resulted in up-regulation of *CycA*, while overexpression of *Polycomb* and *Polyhomeotic* resulted in down-regulation of *CycA*, indicating that action of the PRC1 complex may restrict *CycA* expression. Psc coprecipitated with Pc and Ph, indicating that it belongs to the PRC1 complex [9], [47], [48]. The finding that PRC1 represses expression of CycA is consistent with our finding that Psc and Su(z)2 serve as tumor suppressors in the CySC lineage. Surprisingly, we did not observe massive overproliferation in mutant clones lacking function of PRC1 subunit *Pc* in the CySC lineage (data not shown), unlike as previously reported for wing discs [49], [50] suggesting that a different PRC1 complex might be at play in the CySC lineage.

The role of PcG proteins in regulating proliferation is conserved in mammals. *Mel18* and *BMI-1*, two homologues of *Psc* and *Su(z)2* [7], [8], [9], have been implicated in cell cycle control. As in the case of *Psc* and *Su(z)2*, loss of *Mel18* function results in tumorigenesis, indicating that it also acts as a tumor suppressor [13], [14], [16], [17]. Conversely, *BMI-1* is highly expressed in a number of known cancers and, unlike *Psc* and *Su(z)2* as reported in this work, overexpression of *BMI-1* in mice results in the development of lymphoma [51], [52].

### Dispensability of Psc and Su(z)2 in the germline

Psc and Su(z)2 appeared to be dispensable in the male germline for repression of *Abd-B*, maintenance of GSCs, and germ cell differentiation. Likewise, Psc and Su(z)2 and the PcG proteins Enhancer of Polycomb, Additional sex combs, Polycomb-like, Polycomb, and Extra sexcombs, did not seem to be required for the development of the female germline in *Drosophila* [53], [54]. Although these PcG proteins are actively transcribed in the female germline, loss of function did not impair oocyte development. Rather, function of PcG components is required during the early development of the embryo. It is possible that *l(3)73Ah*, a highly similar *Drosophila* homologue of *Psc* and *Su(z)2* expressed in testis, may substitute functionally for Psc and Su(z)2 in male germ cells [8]. Alternatively, Psc and Su(z)2 may function in a different complex or pathway in the CySC and GSC lineages. For example, while Psc and Su(z)2 are redundant in the CySC lineage and wing discs, embryos lacking members of the PRC1 complex such as Psc, and Su(z)2 were not phenotypically identical [49], [50], [54]. Similarly, different PRC1 complexes may be present in the CySC and GSC lineages, perhaps targeting different loci in each cell type. For example, Pc mutant clones had a growth phenotype in the fly wing disc [49], [50] but not in the testis (data not shown).

### GSCs are displaced by tumorigenesis in the CySC lineage

Our work may provide a mechanism to explain how resident stem cells can be displaced from their niche by tumor cells. Tumorigenesis in the CySC lineage caused by the loss of Psc and Su(z)2 interfered with GSC maintenance non-cell autonomously, apparently by displacing GSCs from their niche. The loss of *Psc* and *Su(z)2* function in the CySC lineage resulted in overexpression of cell adhesion molecules Fas3 and E-Cad, both of which are expressed highly in hub cells but not in GSCs. If the high levels of these cell adhesion molecules in the *Psc* and *Su(z)2* mutant tumor cells results in high affinity for the hub, it may allow the tumor cells to out compete GSCs for adhesion to the hub, thereby displacing GSCs from their niche. Similar effects may underlie the propensity of metastatic cancer cells to displace non-tumor resident stem cells from a niche critical for the survival of normal, resident stem cells. For example, in mammals, prostate cancer cells frequently metastasize to bone marrow, where they occupy the niche of resident hematopoietic stem cells, impairing their function [55].

## Supporting Information

Figure S1 **C587-GAL4 also drives expression of transgenes in the germline but at a low frequency.** Analysis of testes with clones induced in the CySC (*C587-GAL4;FRT42D, Df(2R)Su(z)2^1.b8^/FRT42D, ubi-nGFP;UAS-FLP/tub-GAL80^ts^*) and control (*C587-GAL4;FRT42D/FRT42D, ubi-nGFP;UAS-FLP/tub-GAL80^ts^*). Percentage of testes showing FRT42D controls (blue line) and *Psc* and *Su(z)2* double mutant (red line) germline clones.
(TIF)

Figure S2 ***Psc*** **or** ***Su(z)2*** **are not each required cell-autonomously as tumor suppressors or for GSC maintenance.** (**A–C**): Analysis of *Psc* and *Su(z)2* double mutant germline clones at different stages (*hs-FLP;FRT42D, Df(2R)Su(z)2^1.b8^/FRT42D, ubi-nGFP*). Spermatogonia (A–A'), spermatocyte (B–B'), and spermatid clones (C–C') lacking *Psc* and *Su(z)2* marked by absence of nGFP (dashed line). Phase (A, B, C) and nGFP (A, B, C). (**D–F'**): Analysis of FRT42D control (D, D'; *hs-FLP;FRT42D/FRT42D, ubi-nGFP*), *Psc* mutant (E, E'; *hs-FLP;FRT42D, Psc^e24^/FRT42D, ubi-nGFP*), and *Su(z)2* mutant (F, F';*hs-FLP;FRT42D, Su(z)2^1.b7^/FRT42D, ubi-nGFP*) clones generated by heat shock in both the GSC and CySC lineage. Germline clones marked by the absence of nGFP (D', E', F', arrows). No mutant cell aggregates were observed at the tip of the testes. Phase images (D, E, F). (**G**): Percentage of testes with *Psc* (red line), *Su(z)2* (yellow line) or FRT42D control (blue line) spermatocyte cyst clones at different time points after clone induction. Data reported as average +/− S.D. Scale bars: 50 μm.
(TIF)

Figure S3 ***Psc*** **and** ***Su(z)2*** **double mutant cell aggregates increase in size over time.** (**A**): Size of *Psc-Su(z)2* double mutant aggregates at indicated days after clonal induction (*hs-FLP;FRT42D, Df(2R)Su(z)2^1.b8^/FRT42D, ubi-nGFP*). Data reported as average total pixels of mutant aggregates +/− S.D. It is important to note that testes contain mutant aggregates that originated from one or more mutant clones generated during heat shock, which explains the large deviation in overall size.
(TIF)

Figure S4 **Normal expression of markers of the CySC lineage and hub.** (**A–I'**): Immunostain of FRT42D wild-type control testes (*hs-FLP;FRT42D/FRT42D, ubi-nGFP*). 8 days after clone induction by heat shock. Testis immunostained with anti-GFP (green), anti-Vasa (A–A'), anti-Zfh-1 (B–B'), anti-Eya (C–C'), anti-Tj (D–D'), anti-Fas3 (E–E'), anti-Arm (F–F'), anti-E-Cad (G–G'), anti-Cactus (H–H') and anti-N-Cad (I–I') antibodies. Scale bars: 50 μm.
(TIF)

Figure S5 **Loss of** ***Psc*** **and** ***Su(z)2*** **in the germline did not result in derepression of** ***Abd-B.*** (**A–D’’**) Immunofluorescence analysis of testes with heat shock induced *Psc* and *Su(z)2* mutant clones (*hs-FLP;FRT42D, Df(2R)Su(z)2^1.b8^/FRT42D, ubi-nGFP*). Testes immunostained with anti-GFP (A, B, C, D, green), anti-ABD-B (A–D’’, white or blue), and anti-Vasa (A’’, B, C, D, red). Abnormal somatic mutant cell aggregates (white dashed line); (yellow dashed line) mutant germline clones. (B’’, C’’, D’’) Same area of the testis imaged in the plane of the sheath cells. Arrows mark sheath cell nuclei. Scale bar: 50 µm in A–A’’ and 10 µm in B–D’’. (**E–F’’**): Analysis of testes after 12 days of (E–E’’) simultaneous RNAi knockdown of both *Psc* and *Su(z)2* in the germline (*nanos-GAL4;UAS-Psc-RNAi/UAS-Su(z)2-RNAi*). (F–F’’) Control testes with single RNAi knockdown *Psc* (*nanos-GAL4;UAS-Psc-RNAi/CyO*) grown under the same conditions. Testes immunostained with anti-ABD-B (E, F, white), anti-Vasa (E’, F’, red), anti-TJ (E’’, F’’, green). Scale bar: 50 µm.
(TIF)

Figure S6 ***lines*** **mutant clones share some but not all characteristics of** ***Psc*** **and** ***Su(z)2*** **double mutant clones.** (**A–E’’’’**): Immunostain of testes 8 days after induction of *lines^G2^* mutant clones by heat shock (*hs-FLP;FRT42D, lines^G2^/FRT42D, ubi-nGFP*). Testes with somatic *lines^G2^* mutant clones (dashed line) immunostained with anti-GFP (green), DAPI (blue), (A') anti-Zfh-1, (A’’) anti-Eya, (B') anti-Tj, (B’’) anti-E-Cad, (C') anti-Arm, (C’’, E’’) anti-vasa, (D') anti-Fas3, (D’’) anti-N-Cad, and (E') anti-ABD-B antibodies. Scale bars: 50 μm.
(TIF)
