# Supplementary figures and images for: Polycomb Group Genes Psc and Su(z)2 Maintain Somatic Stem Cell Identity and Activity in Drosophila
Source: PLoS One. 2012 Dec 21;7(12):e52892. doi: 10.1371/journal.pone.0052892 (PMC3528704; doi:10.1371/journal.pone.0052892)

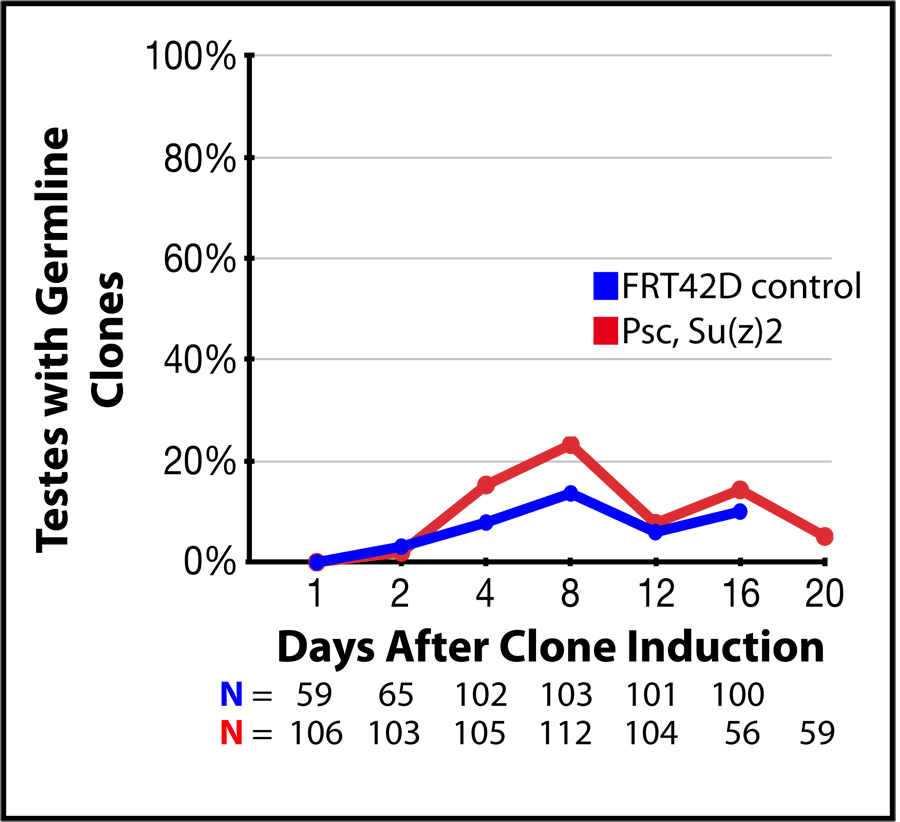

Supplement: Figure S1 — C587-GAL4 also drives expression of transgenes in the germline but at a low frequency. Analysis of testes with clones induced in the CySC (C587-GAL4;FRT42D, Df(2R)Su(z)21.b8/FRT42D, ubi-nGFP;UAS-FLP/tub-GAL80ts) and control (C587-GAL4;FRT42D/FRT42D, ubi-nGFP;UAS-FLP/tub-GAL80ts). Percentage of testes showing FRT42D controls (blue line) and Psc and Su(z)2 double mutant (red line) germline clones. (TIF) [file pone.0052892.s001.tif]

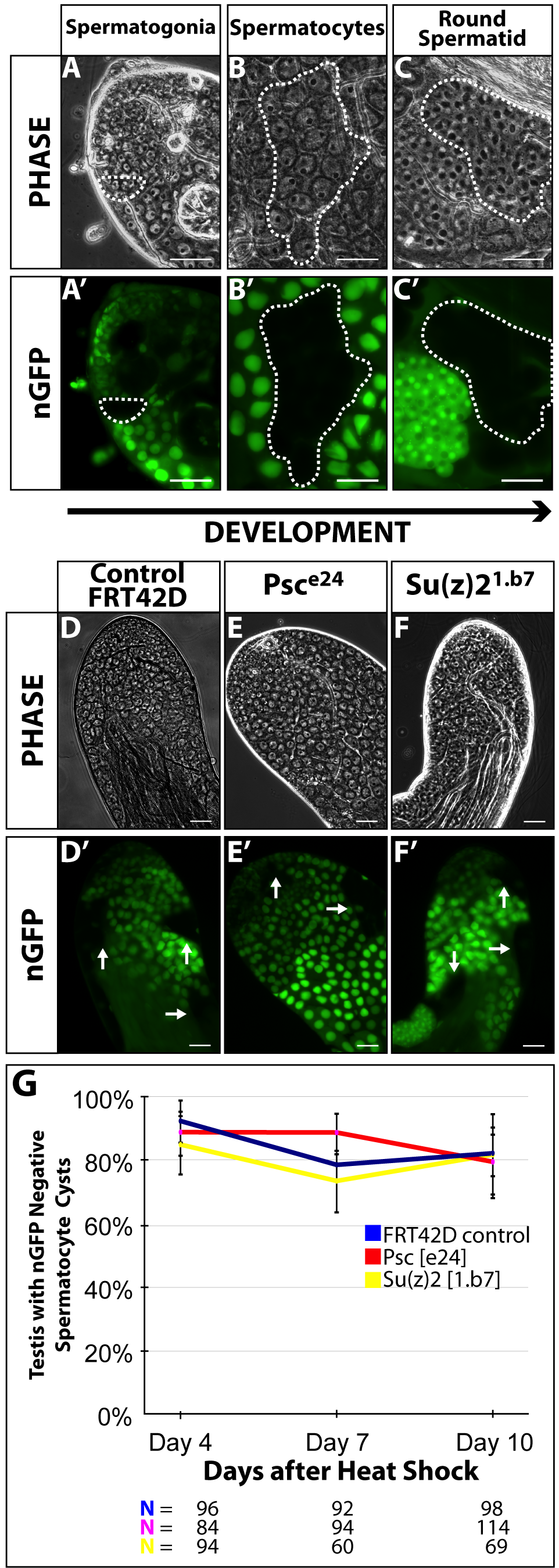

Supplement: Figure S2 — Psc or Su(z)2 are not each required cell-autonomously as tumor suppressors or for GSC maintenance. (A–C): Analysis of Psc and Su(z)2 double mutant germline clones at different stages (hs-FLP;FRT42D, Df(2R)Su(z)21.b8/FRT42D, ubi-nGFP). Spermatogonia (A–A'), spermatocyte (B–B'), and spermatid clones (C–C') lacking Psc and Su(z)2 marked by absence of nGFP (dashed line). Phase (A, B, C) and nGFP (A, B, C). (D–F'): Analysis of FRT42D control (D, D'; hs-FLP;FRT42D/FRT42D, ubi-nGFP), Psc mutant (E, E'; hs-FLP;FRT42D, Psce24/FRT42D, ubi-nGFP), and Su(z)2 mutant (F, F';hs-FLP;FRT42D, Su(z)21.b7/FRT42D, ubi-nGFP) clones generated by heat shock in both the GSC and CySC lineage. Germline clones marked by the absence of nGFP (D', E', F', arrows). No mutant cell aggregates were observed at the tip of the testes. Phase images (D, E, F). (G): Percentage of testes with Psc (red line), Su(z)2 (yellow line) or FRT42D control (blue line) spermatocyte cyst clones at different time points after clone induction. Data reported as average +/− S.D. Scale bars: 50 μm. (TIF) [file pone.0052892.s002.tif]

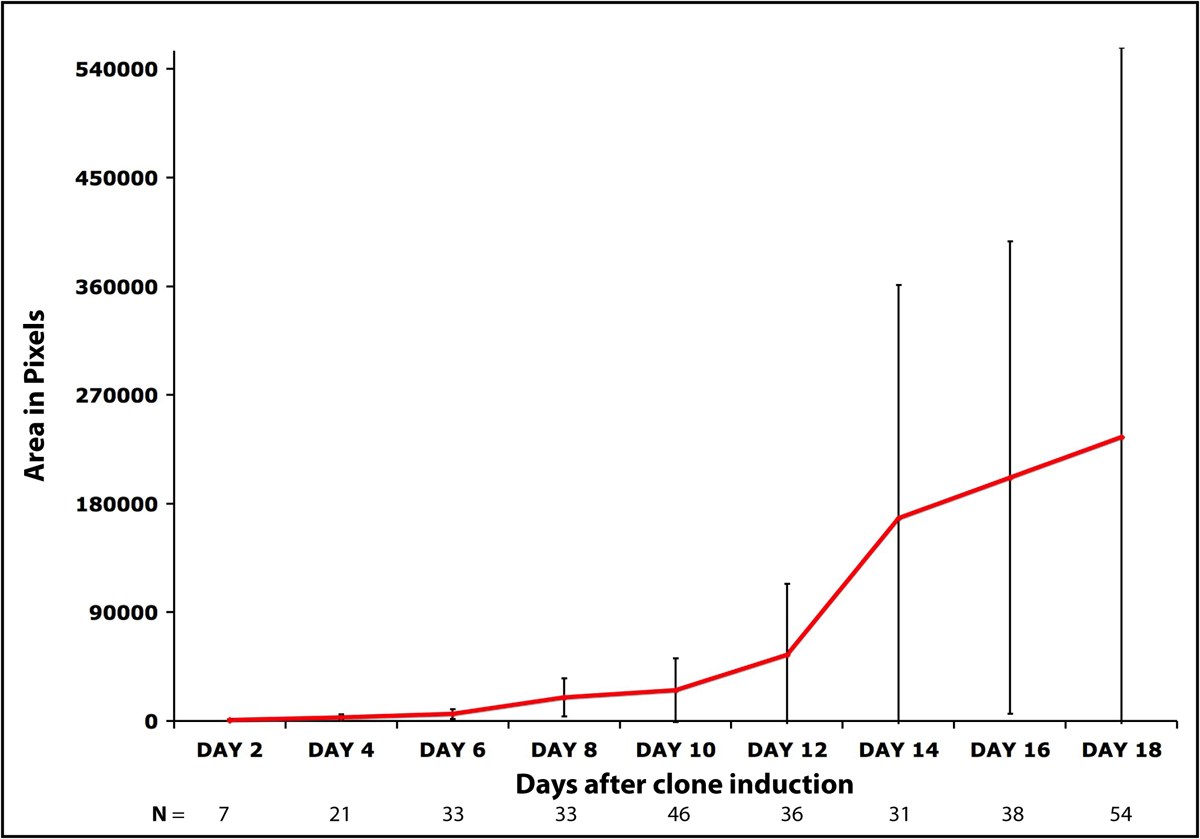

Supplement: Figure S3 — Psc and Su(z)2 double mutant cell aggregates increase in size over time. (A): Size of Psc-Su(z)2 double mutant aggregates at indicated days after clonal induction (hs-FLP;FRT42D, Df(2R)Su(z)21.b8/FRT42D, ubi-nGFP). Data reported as average total pixels of mutant aggregates +/− S.D. It is important to note that testes contain mutant aggregates that originated from one or more mutant clones generated during heat shock, which explains the large deviation in overall size. (TIF) [file pone.0052892.s003.tif]

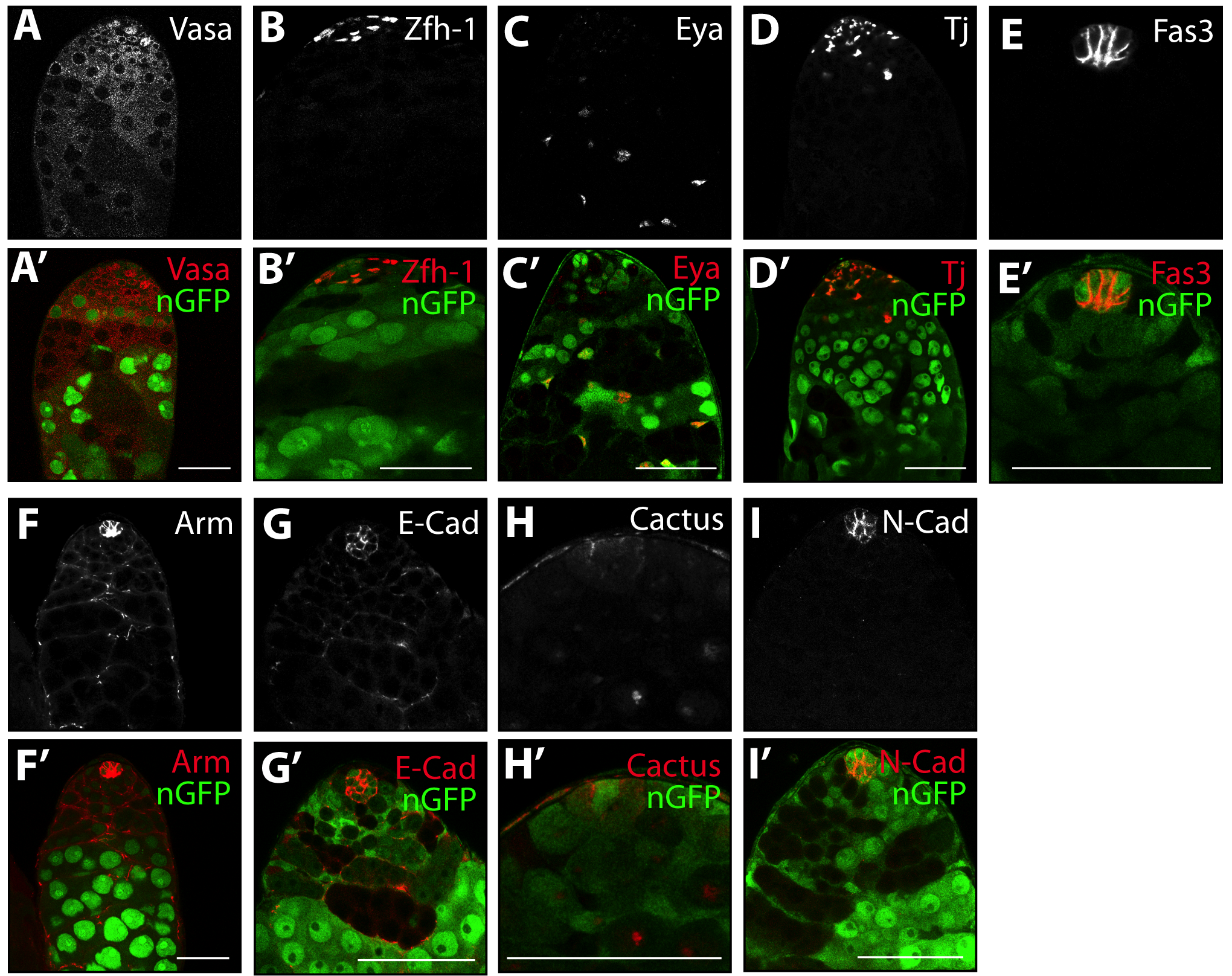

Supplement: Figure S4 — Normal expression of markers of the CySC lineage and hub. (A–I'): Immunostain of FRT42D wild-type control testes (hs-FLP;FRT42D/FRT42D, ubi-nGFP). 8 days after clone induction by heat shock. Testis immunostained with anti-GFP (green), anti-Vasa (A–A'), anti-Zfh-1 (B–B'), anti-Eya (C–C'), anti-Tj (D–D'), anti-Fas3 (E–E'), anti-Arm (F–F'), anti-E-Cad (G–G'), anti-Cactus (H–H') and anti-N-Cad (I–I') antibodies. Scale bars: 50 μm. (TIF) [file pone.0052892.s004.tif]

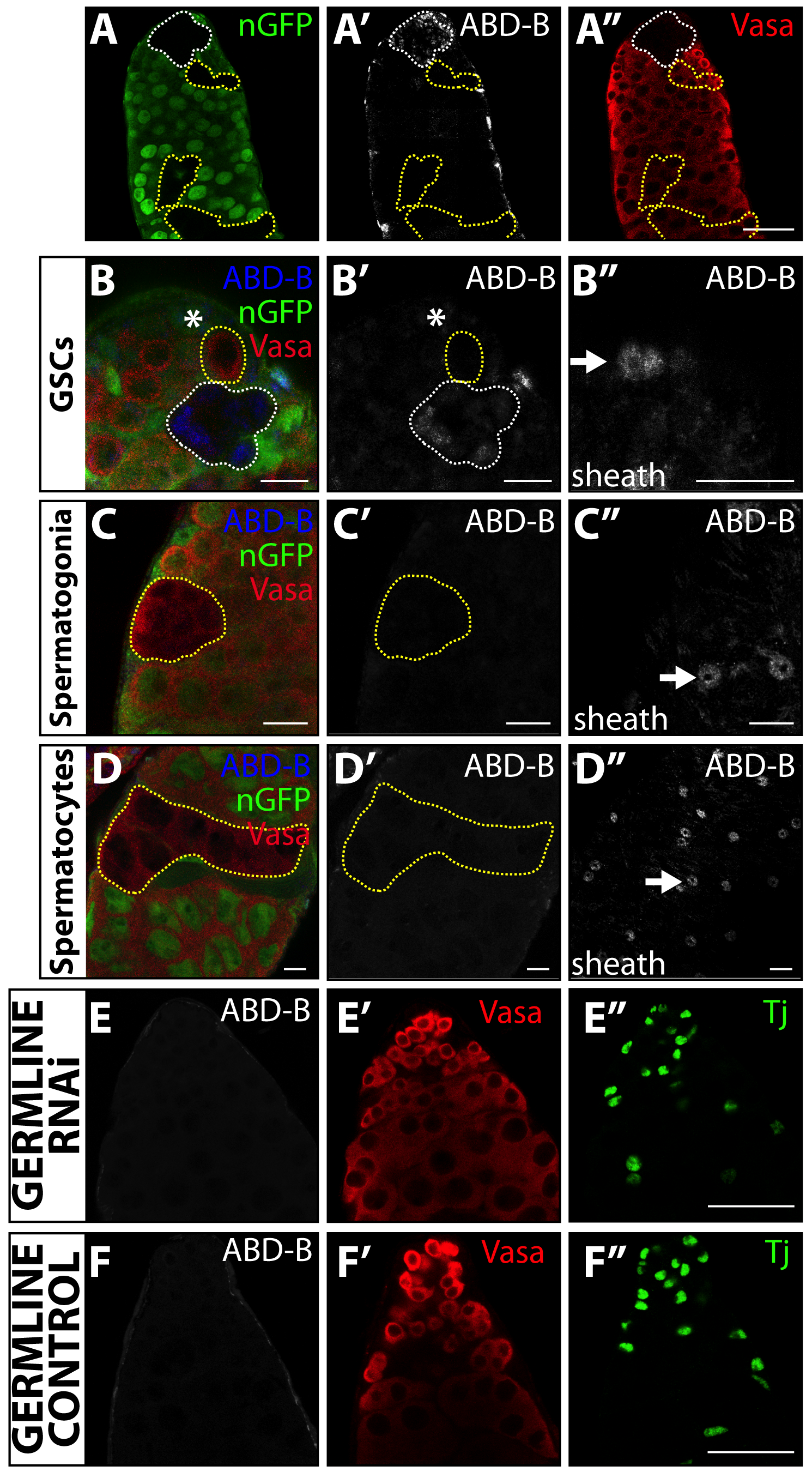

Supplement: Figure S5 — Loss of Psc and Su(z)2 in the germline did not result in derepression of Abd-B. (A–D’’) Immunofluorescence analysis of testes with heat shock induced Psc and Su(z)2 mutant clones (hs-FLP;FRT42D, Df(2R)Su(z)21.b8/FRT42D, ubi-nGFP). Testes immunostained with anti-GFP (A, B, C, D, green), anti-ABD-B (A–D’’, white or blue), and anti-Vasa (A’’, B, C, D, red). Abnormal somatic mutant cell aggregates (white dashed line); (yellow dashed line) mutant germline clones. (B’’, C’’, D’’) Same area of the testis imaged in the plane of the sheath cells. Arrows mark sheath cell nuclei. Scale bar: 50 µm in A–A’’ and 10 µm in B–D’’. (E–F’’): Analysis of testes after 12 days of (E–E’’) simultaneous RNAi knockdown of both Psc and Su(z)2 in the germline (nanos-GAL4;UAS-Psc-RNAi/UAS-Su(z)2-RNAi). (F–F’’) Control testes with single RNAi knockdown Psc (nanos-GAL4;UAS-Psc-RNAi/CyO) grown under the same conditions. Testes immunostained with anti-ABD-B (E, F, white), anti-Vasa (E’, F’, red), anti-TJ (E’’, F’’, green). Scale bar: 50 µm. (TIF) [file pone.0052892.s005.tif]

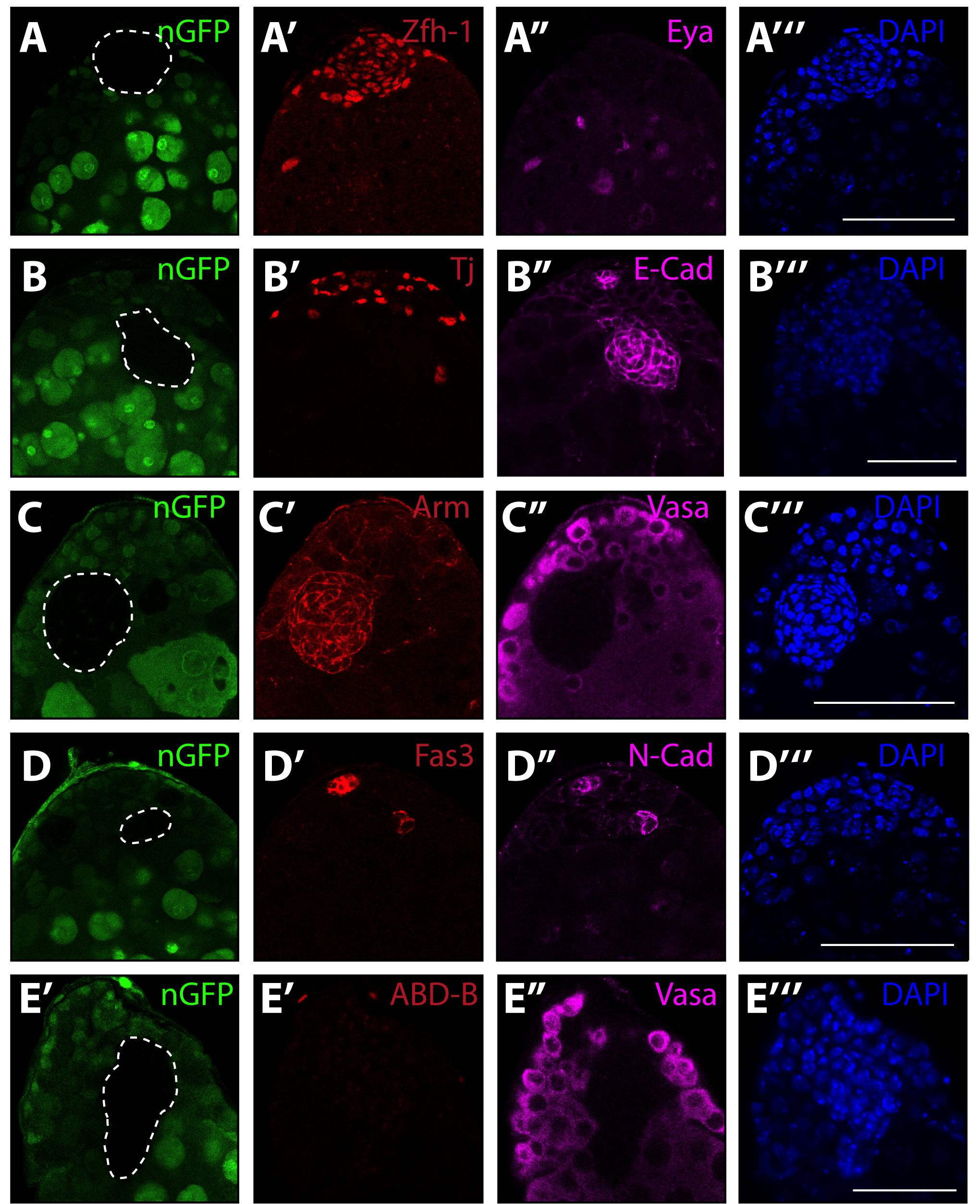

Supplement: Figure S6 — lines mutant clones share some but not all characteristics of Psc and Su(z)2 double mutant clones. (A–E’’’’): Immunostain of testes 8 days after induction of linesG2 mutant clones by heat shock (hs-FLP;FRT42D, linesG2/FRT42D, ubi-nGFP). Testes with somatic linesG2 mutant clones (dashed line) immunostained with anti-GFP (green), DAPI (blue), (A') anti-Zfh-1, (A’’) anti-Eya, (B') anti-Tj, (B’’) anti-E-Cad, (C') anti-Arm, (C’’, E’’) anti-vasa, (D') anti-Fas3, (D’’) anti-N-Cad, and (E') anti-ABD-B antibodies. Scale bars: 50 μm. (TIF) [file pone.0052892.s006.tif]
